# Sequencing and description of the genome of a strain of *Stenotrophomonas geniculata* isolated from a patient infected with COVID-19 at Hospital Regional No.1 de Charo, Michoacán, México

**DOI:** 10.17912/micropub.biology.001051

**Published:** 2024-02-12

**Authors:** Luis Jose Flores-Alvarez, Irma Martínez-Flores, Patricia Bustos, Anel Gómez-García, Sergio Gutiérrez-Castellanos, Augusto César Poot-Hernández, Marina Arredondo-Santoyo

**Affiliations:** 1 Centro de Investigación Biomédica de Michoacán-IMSS. Morelia, Michoacán, México, Mexican Social Security Institute, Mexico City, Mexico City, Mexico; 2 Centro de Ciencias Genomicas, UNAM. Cuernavaca, Morelos, Universidad Nacional Autónoma de México, Mexico City, Mexico City, Mexico; 3 Centro de Investigación Biomédica de Michoacán-IMSS. Morelia, Michoacán, México. , Mexican Social Security Institute, Mexico City, Mexico City, Mexico; 4 Instituto de Fisiología Celular, UNAM. Cd de México, Universidad Nacional Autónoma de México, Mexico City, Mexico City, Mexico

## Abstract

*Stenotrophomonas* is a bacterial genus that can be found in various environments, such as water, soil, and clinical samples. Due to their high genetic and phenotypic diversity, it is difficult to properly identify and classify all isolates. The COVID-19 pandemic caused an increase in nosocomial infections, which played a major role in the high mortality rate among patients in intensive care. This is the first report of the identification of *S. geniculata* as a nosocomial opportunistic pathogen isolated from a patient with COVID-19. Their genome was isolated, sequenced, and assembled, and it consists of 4,488,090 bp in 24 contigs, 4,103 coding sequences, and a G+C content of 66.58%.

**Figure 1. Genome annotations graph and Phylogenetic tree based on the 1479 core genes of each genome:**
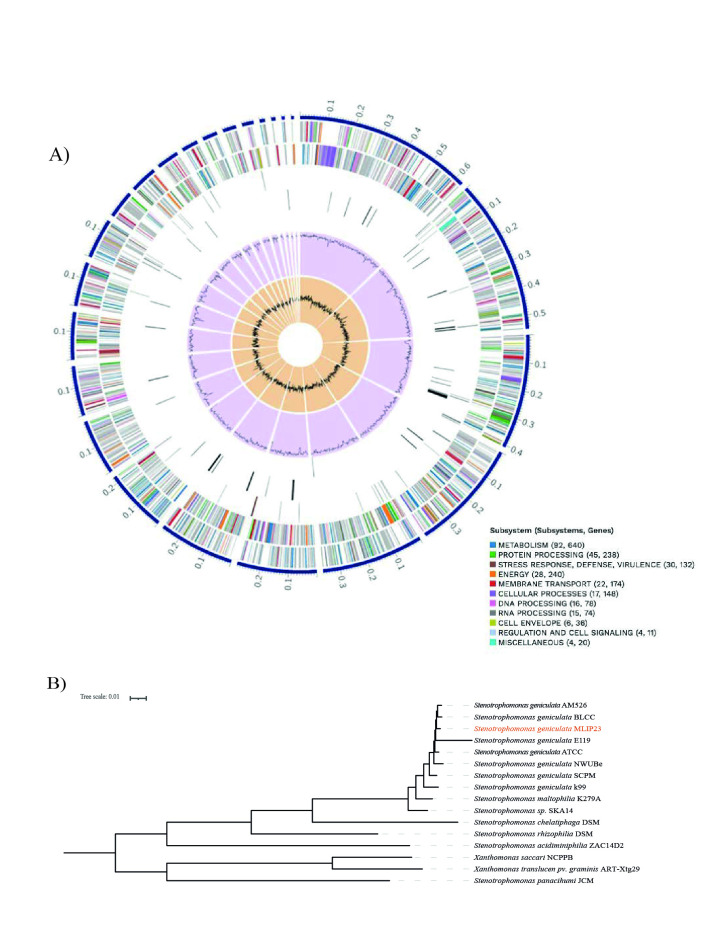
A) Genome annotation graph. The graph shows the annotations of the assembled genome of *Stenotrophomonas* MLIP23 using the comprehensive genome analysis service of the Bacterial and Viral Bioinformatics Resource Center (BVBRC. https://www.bv-brc.org/) (Wattam et al., 2017). The analyses are performed considering annotation statistics and comparison with other genomes in BVBRC within this same species. The annotation graph shows, starting from the outer rings to the inner ones, 24 contigs, CDS in the forward strand, CDS in the reverse strand, RNA genes, CDS with homology to known antimicrobial resistance genes, CDS with homology to known virulence factors, GC content (66.6%) and at the end the GC skew. The CDS colors on the forward and reverse strands indicate the subsystems to which these genes belong. B) Phylogenetic tree. Phylogenetic tree based on the 1479 core genes of families determined with BPGA (Chaudhari et al., 2016), corresponding to the most representative reference genomes, to generate the support in the tree and to determine the phylogenetic placement of MLIP23 genome; which was located in the *S. geniculata* branch.

## Description

The coronavirus is a diverse group of viruses that infect different types of animals (Hu et al., 2021) . At the end of December 2019, several health units in the city of Wuhan, China, reported a group of patients with pneumonia of unknown cause (Wu et al., 2020) . The RNA sequencing of this virus resulted in a new betacoronavirus not previously identified (Wu et al., 2020; Zhu et al., 2020) . This new, highly communicable disease became known as coronavirus disease 2019 (COVID-19) (Wu et al., 2020; Hui et al., 2020) . Due to the COVID-19 pandemic, a percentage of these patients required care treatment in the intensive care unit (ICU) for invasive ventilation (Maes et al., 2021) . Nosocomial infections were common in patients with very severe complications from COVID-19 who were in the ICU, and there seemed to be a connection due to very long stays in that unit (Bardi et al., 2021) . Nosocomial diseases have a special place in hospital clinics due to their importance as one of the main causes of morbidity and mortality. The highest prevalence of nosocomial infections occurs in ICU, surgical, and orthopedic wards, being the most vulnerable population (Lisboa and Rello 2008) . The nosocomial diseases in COVID-19 patients exhibited different groups of Gram-positive ( *Clostridium sp, Staphylococcus aureus* ) and Gram-negative bacteria ( *Escherichia coli, Proteus, Klebsiella, Enterobacter, Serratia marcescens, Pseudomonas spp, Burkholderia cepacia, Stenotrofomonas maltofilia* ) (Lisboa and Rello 2008) .

Bacteria are classified and identified to distinguish between strains according to the criteria of interest to microbiologists and scientists, with species identification being the most important level of classification. Usually, the identification and verification of species involves biochemical, phenotypic criteria, and sometimes serological evaluations. However, currently, genetic criteria are necessary to define a species, particularly when dealing with atypical, rare, or recently described strains (Baron 1996) .

The genus Stenotrophomonas is characterized by having at least 18 recognized species. These bacteria are found in various habitats such as soil, water, associated with plants, animals, and the human body. They are Gram-negative bacteria, morphologically uniform (individual cells or in pairs that are slightly elongated and curved), and they do not form endospores. Generally, they can have one or two polar flagella. Stenotrophomonas uses a limited number of carbon sources. Most species of Stenotrophomonas show positive results for oxide and nitrate reductase tests, as well as for citrate, malonate, hydrolysis of gelatin, and Tween 80 (Ghosh et al., 2020) .

The species that have been found to infect humans have been sequenced and they are not highly virulent organisms, its virulence factors are strain-specific, e. g. the strain of *S. maltophilia* K279a possesses an unusual cell density-signaling pathway and mobile genetics elements such as hemagglutinin and hemolysins, this bacteria is truly an opportunistic microorganism for environmental adaptations (Crossman et al., 2008; Hu et al ., 2021) .

*Stenotrophomonas geniculata* , also known as *Pseudomonas geniculata* , forms a lineage that is phylogenetically highly divergent with other strains of *Pseudomonas* . *S. geniculata* have been observed to be more closely related to *Stenotrophomonas* species (Saati-Santamaria et al., 2021). It is a Gram-negative bacillus that does not form spores, is mobile since it has a polar flagellum, and can form extracellular slime. In culture, pleomorphic forms may appear. On meat peptone agar, colonies are circular, convex, entire, smooth, and transparent. On agar slope, there is good growth (Lysenko 1960) . The G+C genomic content the type of strain is 66.2%, and its genomic size is approximately 4.81 Mbp (Saati-Santamaría et al., 2021).

*S.geniculata* had not been described as a pathogen in humans, only *S. maltophili* a and *S. africana* were the species reported to date (Drancourt et al., 1997) . *S. maltophilia* and *S. africana* are opportunistic pathogens with a very high potential to affect health, not only due to their ability to form biofilm (Pompilio et al., 2011; Brooke 2012; Flores-Treviño et al., 2014; Maes et al., 2021), antibiotic resistance genes, as well as potentially mobile genomic regions and coding for pili and fimbriae may contribute to greater resistance to different antimicrobials (Crossman et al., 2008) .

The COVID-19 pandemic led to the emergence of various opportunistic organisms, some already documented in hospitals, and others, like the one described in this study, such as *S. geniculata* . This marks the first reported instance of this species affecting humans. When classification is done by colony morphology and biochemical test there can be inaccuracies that are only revealed when the organism is sequenced. Nowadays, molecular tools enable a more precise identification. In this study, we sequenced and assembled the genome of *Stenotrophomonas geniculata* isolated from a patient infected with COVID-19.

As a result, the genome had a length of 4,488,090 bp in 24 contigs, with 4,103 coding sequences, 66 tRNA, and a G+C content of 66.58%. No plasmids were identified. A total of 3,013 proteins with functional assignments were identified, mainly related to metabolism and protein processing. According to the PATRIC database, 42 antibiotic resistance genes and one virulence factor were identified. The resistance mechanisms included antibiotic inactivation enzymes (APH(3´)-II/APH(3´)-XV, L1 family), antibiotic target in susceptible species (Alr, Ddl, dxr, EF-G, EF-Tu, folA, Dfr, folP, gyrA, gyrB, Iso-tRNA, kasA, MurA, rho, rpoB, rpoC, S10p, S12p), and efflux pumps conferring antibiotic resistance (EmrAB-OMF, EmrAB-TolC, MacA, MacB, MdtABC-TolC, TolC/OpmH).

## Methods

Strain isolation: The strain was isolated from a pharyngeal exudate of a patient with COVID-19, it was identified using the VITEK 2 Compact (Biomérieux) automated bacteria identification system.

Total DNA isolation: Total DNA was obtained from a pure culture which was grown in 5 mL of Luria Bertani broth overnight at 37°C. The pellet was recovered and bacterial genomic DNA extraction was performed using the ZymoBOIMICS DNA Miniprep Kit (ZymoReseach US Cat D4300) according to the manufacturer's instructions.

Genome sequencing: The genome sequencing service was carried out using the services of the Novogene company, according to its protocols, the genomic DNA was randomly fragmented by sonication, the DNA fragments were polished at the ends, A-sequences were added to the ends and ligated with the full-length adapters for Illumina sequencing. It was amplified by PCR with indexed P5 and P7 oligonucleotides. The PCR products as the final construction of the libraries were purified with the AMPure XP system. Libraries were then checked for size distribution using the Agilent 2100 Bioanalyzer (Agilent Technologies, CA, USA) and quantified using real-time PCR (to meet the 3 nM criteria). Qualified libraries are loaded onto Illumina sequencers after mixing according to their effective concentration and expected data volume. The result was a total raw data of 5.8G, raw reads 38767250, effective (%) 99.82, error (%) 0.03, Q20(%) 97.47, Q30(%)93.03, and GC(%) 65.42. The sequencing quality of the raw sequences obtained was analyzed using the FASTQC program (V.0.11.8).

Genome assembly and annotation. After trimming adapters of the reads with Trim Galore v0.6.4 tool, and to ensure the accuracy and reliability of the genome assembly, we assemble readings with two algorithms, MEGAHIT v1.2.8 and SPAdes v3.13.1. The assemblies were compared with the QUAST v5.0.2 tool; according to its metrics, the best assembler was SPAdes. Although both assemblers provide similar values in terms of the complete size of the assembly and percentage of GC, SPAdes showed longer reading contigs and higher N50 (360,976), suggesting higher assemble integrity, so Spades was the selected assembler; thus, the processed reads were assembled on a high-coverage (500 – 600 X), resulting in 24 contigs with a total length of 4,488,090 bp and an average G+C content of 66.58%. The pre-annotation of the genome was obtained from “The Bacterial and Viral Bioinformatics Resource Center” (BV-BRC, https://www.bv-brc.org/) using the default parameters. The website uses RAST tool to pre-annotate the antibiotic-resistance and virulence-related genes (Brettin et al., 2015) . The genome contained 4,103 protein-coding sequences (CDS), 66 transfer RNA (tRNA) genes, and 5 ribosomal RNA (rRNA) genes ( Fig. 1 A, genome annotations graph ). Using the quality tools in BV-BRC, it was determined that the genome is 100% complete (with checkM program), with a Coarse consistency of 99.6 and Fine Consistency of 99.5.

The taxonomic classification. The genome annotation was carried out with proteins coming, on the one hand, from the reference genomes proposed by BV-BRC based on its phylogenetic analysis, and on the other, from the representative genomes obtained with the GTDBTK v2.1.1 program. Using GTDB-Tk version r207 (Pierre et al., 2022) assigning their classification according to their homology to the closest genomes. These genomes were obtained from the National Center for Biotechnology Information (NCBI, https://www.ncbi.nlm.nih.gov/) and their proteins were annotated with Prokka v1.14.6 (prodigal). The information was used to estimate the phylogenetic classification of the studied genome.

Phylogenetic analysis. The BPGA v1.3 tool was used to conduct the phylogenetic analysis with 1479 core genes (Chaudhari et al., 2016) . Briefly, BPGA uses clustering tools with default values, USEARCH ortholog clustering tool with a 50% identity cutoff for ortholog clustering of genomes, and with the proteins in fasta format, we were able to identify orthologous groups of functional genes. To visualize the phylogeny, inference of a phylogenetic tree ( Fig. 1 B ) using the maximum likelihood method was performed using the phylogenetic software package IQ-TREE v2.1.2 with the best parameters (1000 bootstrap replicates and LG+F+R7 model; Chernomor et al., 2016; Nguyen et al., 2015). Briefly, IQ-TREE automatically detects the sequence type and performs a rapid selection of the best-fitting replacement model via ModelFinder (Kalyaanamoorthy et at., 2017) , an effective tree search algorithm (Chernomor et al., 2016) , and a Bootstrap approximation (Minh et al., 2014; Hoang et al., 2018) . To draw and edit the phylogenetic trees, the Interactive Tree Of Life program (iTOL v6.6) was used (Letunic et al., 2019) . The analysis showed that all strains used were closely related, and it could be estimated that local strain belongs to *Stenotrophomonas geniculata* , with a 98% identity and over 90% coverage when compared to clones from the same branch ( Figure 1 B ).

## Reagents

This whole-genome shotgun project has been deposited at GenBank. IBID SUB13887264, Bioproject PRJNA1025098, BioSample SAMN37712844. Organism *Stenotrophomonas geniculata* . Sample: MLIP23.
